# A nationwide analysis on the interaction between frailty and beta-blocker therapy in hip fracture patients

**DOI:** 10.1007/s00068-023-02219-7

**Published:** 2023-01-12

**Authors:** Maximilian Peter Forssten, Ahmad Mohammad Ismail, Ioannis Ioannidis, Per Wretenberg, Tomas Borg, Yang Cao, Marcelo A. F. Ribeiro, Shahin Mohseni

**Affiliations:** 1 Department of Orthopedic Surgery, Orebro University Hospital, 701 85 Orebro, Sweden; 2 Division of Trauma and Emergency Surgery, Department of Surgery, Orebro University Hospital, 701 85 Orebro, Sweden; 3 School of Medical Sciences, Orebro University, 702 81 Orebro, Sweden; 4 Clinical Epidemiology and Biostatistics, School of Medical Sciences, Orebro University, 701 82 Orebro, Sweden; 5 Pontifical Catholic University of São Paulo, São Paulo, Brazil; 6 Trauma, Burns, Critical Care and Acute Care Surgery, Department of Surgery, Sheikh Shakhbout Medical City, Mayo Clinic, Abu Dhabi, United Arab Emirates

**Keywords:** Hip fracture, Frailty, Beta-blocker therapy, Orthopedic Hip Frailty Score, Mortality, Inverse probability of treatment weighting

## Abstract

**Introduction:**

Hip fracture patients, who are often frail, continue to be a challenge for healthcare systems with a high postoperative mortality rate. While beta-blocker therapy (BBt) has shown a strong association with reduced postoperative mortality, its effect in frail patients has yet to be determined. This study’s aim is to investigate how frailty, measured using the Orthopedic Hip Frailty Score (OFS), modifies the effect of preadmission beta-blocker therapy on mortality in hip fracture patients.

**Methods:**

This retrospective register-based study included all adult patients in Sweden who suffered a traumatic hip fracture and subsequently underwent surgery between 2008 and 2017. Treatment effect was evaluated using the absolute risk reduction (ARR) in 30-day postoperative mortality when comparing patients with (BBt+) and without (BBt-) ongoing BBt. Inverse probability of treatment weighting (IPTW) was used to reduce potential confounding when examining the treatment effect. Patients were stratified based on their OFS (0, 1, 2, 3, 4 and 5) and the treatment effect was also assessed within each stratum.

**Results:**

A total of 127,305 patients were included, of whom 39% had BBt. When IPTW was performed, there were no residual differences in observed baseline characteristics between the BBt+ and BBt- groups, across all strata. This analysis found that there was a stepwise increase in the ARRs for each additional point on the OFS. Non-frail BBt+ patients (OFS 0) exhibited an ARR of 2.2% [95% confidence interval (CI) 2.0–2.4%, *p* < 0.001], while the most frail BBt+ patients (OFS 5) had an ARR of 24% [95% CI 18–30%, *p* < 0.001], compared to BBt- patients within the same stratum.

**Conclusion:**

Beta-blocker therapy is associated with a reduced risk of 30-day postoperative mortality in frail hip fracture patients, with a greater effect being observed with higher Orthopedic Hip Frailty Scores.

**Supplementary Information:**

The online version contains supplementary material available at 10.1007/s00068-023-02219-7.

## Introduction

Hip fracture patients comprise a significant challenge for health care systems globally. Owing to the high degree of frailty and considerable comorbidity burden in this population, they demand a substantial amount of resources both during their hospital stay and post discharge [1–3]. In 2010, an estimated 2.7 million people suffered a hip fracture, accounting for approximately 20% of all osteoporotic fractures in those who were 50 years or older [4–6]. This number is projected to continue to rise as the global population ages and life expectancy increases [7, 8]. With 6.3 million hip fractures yearly being predicted by 2050 [9], an even greater strain will be placed on hospital and public resources, necessitating a robust strategy to manage this increased demand [1, 10–13]. However, hip fracture patients are a heterogenous population, wherein specific subgroups are disproportionately culpable for the high mortality rates observed [14–17].

Frailty plays a particular role in the high rates of postoperative mortality observed in hip fracture patients, with up to 27% of patients dying within the first year after surgery [15, 17]. Frailty, defined as a reduced ability to adapt to external stressors due to a reduced physiological reserve [18–21], is relatively common in hip fracture patients; rates vary between one fifth and half of hip fracture patients depending on the method used for measuring it [22–26]. Nevertheless, there is a distinct lack of tools tailored to helping this vulnerable cohort, once a patient has been identified as frail [27, 28]. Besides selection of operative technique, when fracture morphology allows for it [29, 30], the chief potential intervention has been orthogeriatric care models [31–33]. However, these models tend to include patients based on chronological age, rather than biological age or degree of frailty, which might not always be optimal [34–37]. Consequently, more options are required in order to adapt care to those who are most frail.

Beta-blocker therapy (BBt) has previously been associated with reduced mortality in hip fracture patients, both in the short and long term [38–42]. The proposed mechanism of action being that BBt inhibits pathways that otherwise mediate the hyperadrenergic response that stems from the initial trauma and subsequent surgical interventions [43, 44]. Given the inherent nature of frailty, it can be suspected that frail patients may be more susceptible to the adverse effects of this hyperadrenergic state, considering their reduced physiologic reserve compared to more robust patients. Subsequently, BBt should also be associated with a greater reduction in mortality the more frail a hip fracture patient is. The aim of the current study was therefore to investigate how frailty, measured using the Orthopedic Hip Frailty Score (OFS), modifies the effect of preadmission beta-blocker therapy on mortality in hip fracture patients.

## Methods

Data for this retrospective cohort study were obtained from the prospectively collected Swedish National Quality Register for Hip Fractures, Rikshöft [45]. All patients who underwent primary emergency hip fracture surgery in Sweden between 2008 and 2017 were eligible for inclusion. Conservatively managed and pathological hip fracture were excluded from the original data retrieval. Patients were also excluded if they were missing data required to calculate the OFS. Rikshöft provided information relating to patient demographics, admission and discharge, as well as perioperative and operative data. Using the patients’ social security numbers, this dataset was cross-referenced with the Swedish National Board of Health and Welfare’s Patient, Cause of Death, and Prescribed Drugs registers. The National Patient Register is comprised of all ICD-10 code recorded during all periods of in-patient and out-patient care, for each patient [46]. The Cause of Death Register contains the time and cause of death for anyone who dies in Sweden, irrespective of country of residence [47]. The National Prescribed Drug Register records all prescriptions issued by physicians in Sweden at primary and secondary care facilities [48]. This investigation in its entirety adheres to the strengthening the reporting of observational studies in epidemiology (STROBE) guidelines as well as the principles of the Declaration of Helsinki [49]. Ethical approval was secured from the Swedish Ethical Review Authority (reference 2022-03107-02).

### Beta-blocker therapy

The Prescribed Drugs Register is a national Swedish database that logs all drug prescriptions issued by physicians within both primary and secondary care facilities. A retrieval request was made for all beta-blocker prescriptions (ATC codes C07AA, C07AB, C07AG) issued for the previously specified patients registered in Rikshöft. Ongoing BBt was defined as having filled a prescription within the 12-month period prior to, as well as subsequent to, hip fracture surgery. To avoid immortal time bias, patients who filled a prescription preoperatively, but died prior to filling a prescription postoperatively, were counted as having ongoing BBt. This 12-month period was decided on as beta-blockers are rarely discontinued once prescribed and therefore generally prescribed on a long-term basis, often covering up to a one-year period with a single prescription. Preoperative discontinuation of BBt is also rare, with no such events being found when assessing the electronic medical records of 2443 consecutive hip fracture patients operated over a 5-year period in Orebro County, Sweden [38].

### Calculating the Orthopedic Hip Frailty Score

The Orthopedic Hip Frailty Score is a recently validated score for measuring frailty in hip fracture patients [50]. The OFS was determined based on the presence of the five binary variables: an age ≥ 85 years old, congestive heart failure, a history of malignancy (local or metastatic, excluding non-invasive neoplasms of the skin), institutionalization, and a non-independent functional status (i.e., requiring assistance with activities of daily life). A patient received 1 point for each variable present, with the maximum possible score being 5 [50].

### Statistical analysis

Cases were stratified according to their OFS; OFS 0, OFS 1, OFS 2, OFS 3, OFS 4, and OFS 5. Within each stratum, cases were further partitioned based on if they had ongoing beta-blocker therapy (BBt+), or no ongoing beta-blocker therapy (BBt-) at the time of admission. As continuous variables were non-normally distributed, they were summarized as medians and interquartile ranges (IQRs), with the Kruskall–Wallis test being used as an omnibus test. Categorical variables were instead presented as counts and percentages. Either the Chi-squared test or Fisher’s exact test was used, as appropriate, to compare the distribution of categorical variables within the cohorts. The primary outcome of interest was 30-day postoperative mortality.

To account for potential confounding, inverse probability of treatment weighting (IPTW) was used to balance the cohorts within each stratum. The probability of treatment was assessed using a logistic regression model; BBt was set as the dependent variable while the predictors consisted of age, sex, American Society of Anesthesiologists (ASA) classification, type of fracture, type of surgery, previous myocardial infarction, peripheral vascular disease, cerebrovascular disease, diabetes mellitus, dementia, chronic obstructive pulmonary disease, connective tissue disease, liver disease, and chronic kidney disease. The weights were calculated as $\frac{1}{\mathrm{probability \; of \; BBt}+}$ for BBt + patients and $\frac{1}{1-\mathrm{probability \; of \; BBt}+}$ for BBt- patients. After IPTW, balance between the cohorts within each stratum was evaluated by employing absolute standardized differences (ASDs), where an ASD < 0.1 was considered balanced. The absolute risk reduction (ARR) between the BBt+ and BBt- patients, as well as the corresponding confidence interval (CI), were then calculated within each stratum [51]. A sensitivity analysis was also performed where the individual components of the OFS were included in the logistic regression model, to account for differences in the distribution of the component variables of the OFS between the BBt- and BBt+ patients.

Statistical significance was defined as a two-sided *p* value less than 0.05. Among cases included in the analyses, less than two percent had any form of missing data. Data that were missing were consequently assumed to be missing at random. To handle missing data, multiple imputation by chained equations was employed; logistic regression was used for sex, a proportional odds model for ASA classification, as well as Bayesian polytomous regression for type of fracture and type of surgery. Analyses were performed using the tidyverse, haven, parallel, mice, survey, tableone, and writexl packages in the statistical software R 4.0.5 (R Foundation for Statistical Computing, Vienna, Austria) [52].

## Results

After applying the inclusion and exclusion criteria, 127,305 cases remained. In this population, 39% of the patients were in the BBt+ cohorts (*N* = 49,743). Metoprolol was consistently the most common beta-blocker prescribed (BBt+ , OFS 0 vs OFS 5: 56.1% vs 56.6%); however, the proportion who filled a prescription for bisoprolol increased stepwise with the OFS (BBt+ , OFS 0 vs OFS 5: 15.2% vs 26.4%). Patients with a higher OFS were generally older (BBt-, OFS 0 vs OFS 5: 90 years vs 74 years), less fit for surgery according to their ASA classification (BBt-, OFS 0 vs OFS 5: ASA ≥ 3, 26.9% vs 91.9%), and had a higher comorbidity burden according to their Charlson Comorbidity Index (CCI) (BBt-, OFS 0 vs OFS 5: CCI ≥ 7, 1.9% vs 100%). All comorbidities, except for liver disease, increased along with the OFS. The use of all types of arthroplasty decreased as patients became more frail (BBt-, OFS 0 vs OFS 5: 31.9% vs 26.2%), while the proportion of these patients managed using of hemiarthroplasty significantly increased (BBt-, OFS 0 vs OFS 5: 43.9% vs 98.1%) (Table 1).

**Table 1:** Demographics and clinical characteristics of hip fracture patients

	OFS 0		OFS 1		OFS 2		OFS 3		OFS 4		OFS 5		
	BBt- (*N* = 20,003)	BBt+ (*N* = 10,460)	BBt- (*N* = 21,218)	BBt+ (*N* = 14,094)	BBt- (*N* = 20,113)	BBt+ (*N* = 13,685)	BBt- (*N* = 13,187)	BBt+ (*N* = 8672)	BBt- (*N* = 2831)	BBt+ (*N* = 2597)	BBt- (*N* = 210)	BBt+ (*N* = 235)	*p* value
Age, median [IQR]	74 [66–80]	77 [71–81]	82 [76–87]	82 [77–86]	87 [82–90]	86 [82–90]	89 [87–93]	89 [86–92]	90 [87–93]	90 [87–92]	90 [88–94]	89 [87–92]	< 0.001
Sex, *n* (%)													< 0.001
Female	12,857 (64.3)	7058 (67.5)	14,193 (66.9)	9670 (68.6)	13,743 (68.3)	9803 (71.6)	9287 (70.4)	6301 (72.7)	1663 (58.7)	1801 (69.3)	97 (46.2)	130 (55.3)	
Male	7144 (35.7)	3399 (32.5)	7023 (33.1)	4423 (31.4)	6368 (31.7)	3882 (28.4)	3900 (29.6)	2371 (27.3)	1168 (41.3)	795 (30.6)	113 (53.8)	105 (44.7)	
Missing	2 (0.0)	3 (0.0)	2 (0.0)	1 (0.0)	2 (0.0)	0 (0.0)	0 (0.0)	0 (0.0)	0 (0.0)	1 (0.0)	0 (0.0)	0 (0.0)	
Type of beta-blocker, *n* (%)													< 0.001
Metoprolol	0 (0.0)	5871 (56.1)	0 (0.0)	7952 (56.4)	0 (0.0)	7943 (58.0)	0 (0.0)	5153 (59.4)	0 (0.0)	1524 (58.7)	0 (0.0)	133 (56.6)	
Bisoprolol	0 (0.0)	1593 (15.2)	0 (0.0)	2610 (18.5)	0 (0.0)	2615 (19.1)	0 (0.0)	1709 (19.7)	0 (0.0)	653 (25.1)	0 (0.0)	62 (26.4)	
Atenolol	0 (0.0)	1736 (16.6)	0 (0.0)	2038 (14.5)	0 (0.0)	1790 (13.1)	0 (0.0)	1049 (12.1)	0 (0.0)	188 (7.2)	0 (0.0)	25 (10.6)	
Other	0 (0.0)	1260 (12.0)	0 (0.0)	1494 (10.6)	0 (0.0)	1337 (9.8)	0 (0.0)	761 (8.8)	0 (0.0)	232 (8.9)	0 (0.0)	15 (6.4)	
ASA classification, *n* (%)													< 0.001
1	3779 (18.9)	446 (4.3)	1130 (5.3)	211 (1.5)	522 (2.6)	142 (1.0)	188 (1.4)	52 (0.6)	12 (0.4)	10 (0.4)	0 (0.0)	1 (0.4)	
2	10,514 (52.6)	5277 (50.4)	9326 (44.0)	4941 (35.1)	7003 (34.8)	3527 (25.8)	3464 (26.3)	1489 (17.2)	388 (13.7)	262 (10.1)	13 (6.2)	22 (9.4)	
3	5039 (25.2)	4236 (40.5)	9272 (43.7)	7796 (55.3)	10,592 (52.7)	8380 (61.2)	7888 (59.8)	5776 (66.6)	1856 (65.6)	1770 (68.2)	125 (59.5)	156 (66.4)	
4	340 (1.7)	335 (3.2)	1174 (5.5)	969 (6.9)	1720 (8.6)	1443 (10.5)	1488 (11.3)	1246 (14.4)	535 (18.9)	511 (19.7)	64 (30.5)	51 (21.7)	
5	9 (0.0)	2 (0.0)	14 (0.1)	9 (0.1)	25 (0.1)	11 (0.1)	17 (0.1)	10 (0.1)	9 (0.3)	11 (0.4)	4 (1.9)	2 (0.9)	
Missing	322 (1.6)	164 (1.6)	302 (1.4)	168 (1.2)	251 (1.2)	182 (1.3)	142 (1.1)	99 (1.1)	31 (1.1)	33 (1.3)	4 (1.9)	3 (1.3)	
Type of fracture, *n* (%)													< 0.001
Non-displaced cervical (garden 1–2)	3409 (17.0)	1488 (14.2)	2926 (13.8)	1656 (11.7)	2615 (13.0)	1575 (11.5)	1599 (12.1)	945 (10.9)	350 (12.4)	282 (10.9)	23 (11.0)	29 (12.3)	
Displaced cervical (garden 3–4)	7831 (39.1)	4217 (40.3)	8060 (38.0)	5565 (39.5)	7258 (36.1)	5031 (36.8)	4597 (34.9)	2913 (33.6)	949 (33.5)	873 (33.6)	69 (32.9)	76 (32.3)	
Basicervical	649 (3.2)	296 (2.8)	685 (3.2)	416 (3.0)	704 (3.5)	486 (3.6)	490 (3.7)	321 (3.7)	121 (4.3)	83 (3.2)	9 (4.3)	11 (4.7)	
Peritrochanteric (two fragments)	3465 (17.3)	1898 (18.1)	4210 (19.8)	2798 (19.9)	4196 (20.9)	2884 (21.1)	2914 (22.1)	2030 (23.4)	626 (22.1)	582 (22.4)	48 (22.9)	47 (20.0)	
Peritrochanteric (multiple fragments)	2927 (14.6)	1619 (15.5)	3692 (17.4)	2506 (17.8)	3784 (18.8)	2580 (18.9)	2622 (19.9)	1735 (20.0)	579 (20.5)	530 (20.4)	45 (21.4)	50 (21.3)	
Subtrochanteric	1716 (8.6)	936 (8.9)	1638 (7.7)	1144 (8.1)	1547 (7.7)	1123 (8.2)	962 (7.3)	726 (8.4)	206 (7.3)	246 (9.5)	16 (7.6)	22 (9.4)	
Missing	6 (0.0)	6 (0.1)	7 (0.0)	9 (0.1)	9 (0.0)	6 (0.0)	3 (0.0)	2 (0.0)	0 (0.0)	1 (0.0)	0 (0.0)	0 (0.0)	
Type of surgery, *n* (%)													< 0.001
Pins or screws	4811 (24.1)	1953 (18.7)	3580 (16.9)	2039 (14.5)	3268 (16.2)	1966 (14.4)	2170 (16.5)	1260 (14.5)	505 (17.8)	440 (16.9)	37 (17.6)	46 (19.6)	
Screws or pins with sideplate	4696 (23.5)	2397 (22.9)	5618 (26.5)	3570 (25.3)	5436 (27.0)	3679 (26.9)	3890 (29.5)	2534 (29.2)	822 (29.0)	689 (26.5)	64 (30.5)	56 (23.8)	
Intramedullary nail	4106 (20.5)	2329 (22.3)	4637 (21.9)	3263 (23.2)	4772 (23.7)	3397 (24.8)	3155 (23.9)	2300 (26.5)	715 (25.3)	748 (28.8)	54 (25.7)	73 (31.1)	
Hemiarthroplasty	2801 (14.0)	1909 (18.3)	5799 (27.3)	4070 (28.9)	6060 (30.1)	4138 (30.2)	3792 (28.8)	2421 (27.9)	764 (27.0)	689 (26.5)	54 (25.7)	56 (23.8)	
Total hip replacement	3581 (17.9)	1865 (17.8)	1577 (7.4)	1150 (8.2)	564 (2.8)	501 (3.7)	174 (1.3)	155 (1.8)	23 (0.8)	29 (1.1)	1 (0.5)	4 (1.7)	
Missing	8 (0.0)	7 (0.1)	7 (0.0)	2 (0.0)	13 (0.1)	4 (0.0)	6 (0.0)	2 (0.0)	2 (0.1)	2 (0.1)	0 (0.0)	0 (0.0)	
OFS													
Age ≥ 85, *n* (%)	0 (0.0)	0 (0.0)	7472 (35.2)	4910 (34.8)	12,846 (63.9)	8800 (64.3)	12,003 (91.0)	7547 (87.0)	2748 (97.1)	2494 (96.0)	210 (100.0)	235 (100.0)	< 0.001
Congestive heart failure, *n* (%)	0 (0.0)	0 (0.0)	658 (3.1)	1838 (13.0)	1903 (9.5)	3738 (27.3)	2810 (21.3)	4082 (47.1)	1976 (69.8)	2204 (84.9)	210 (100.0)	235 (100.0)	< 0.001
History of malignancy, *n* (%)	0 (0.0)	0 (0.0)	2836 (13.4)	1555 (11.0)	3184 (15.8)	2027 (14.8)	2495 (18.9)	1728 (19.9)	1336 (47.2)	1000 (38.5)	210 (100.0)	235 (100.0)	< 0.001
Institutionalized, *n* (%)	0 (0.0)	0 (0.0)	2169 (10.2)	531 (3.8)	6590 (32.8)	2463 (18.0)	9673 (73.4)	4519 (52.1)	2463 (87.0)	2127 (81.9)	210 (100.0)	235 (100.0)	< 0.001
Non-independent functional status, *n* (%)	0 (0.0)	0 (0.0)	8083 (38.1)	5260 (37.3)	15,703 (78.1)	10,342 (75.6)	12,580 (95.4)	8140 (93.9)	2801 (98.9)	2563 (98.7)	210 (100.0)	235 (100.0)	< 0.001
Charlson Comorbidity Index, *n* (%)													< 0.001
≤ 4	16,566 (82.8)	7506 (71.8)	11,251 (53.0)	6150 (43.6)	7496 (37.3)	3746 (27.4)	2925 (22.2)	1152 (13.3)	0 (0.0)	0 (0.0)	0 (0.0)	0 (0.0)	
5–6	3051 (15.3)	2450 (23.4)	7662 (36.1)	5615 (39.8)	8986 (44.7)	6193 (45.3)	6857 (52.0)	4218 (48.6)	1090 (38.5)	889 (34.2)	0 (0.0)	0 (0.0)	
≥ 7	386 (1.9)	504 (4.8)	2305 (10.9)	2329 (16.5)	3631 (18.1)	3746 (27.4)	3405 (25.8)	3302 (38.1)	1741 (61.5)	1708 (65.8)	210 (100.0)	235 (100.0)	
Hypertension, *n* (%)	4464 (22.3)	5326 (50.9)	6102 (28.8)	7780 (55.2)	6245 (31.0)	7446 (54.4)	4025 (30.5)	4737 (54.6)	1221 (43.1)	1475 (56.8)	121 (57.6)	165 (70.2)	< 0.001
Arrhythmia, *n* (%)	825 (4.1)	2142 (20.5)	1692 (8.0)	4037 (28.6)	2371 (11.8)	4574 (33.4)	2060 (15.6)	3445 (39.7)	881 (31.1)	1328 (51.1)	101 (48.1)	153 (65.1)	< 0.001
Previous myocardial infarction, *n* (%)	168 (0.8)	600 (5.7)	443 (2.1)	1208 (8.6)	747 (3.7)	1629 (11.9)	723 (5.5)	1240 (14.3)	278 (9.8)	470 (18.1)	26 (12.4)	48 (20.4)	< 0.001
Cerebrovascular disease, *n* (%)	1665 (8.3)	1352 (12.9)	3228 (15.2)	2793 (19.8)	3545 (17.6)	3098 (22.6)	2579 (19.6)	2196 (25.3)	697 (24.6)	740 (28.5)	47 (22.4)	73 (31.1)	< 0.001
Peripheral vascular disease, *n* (%)	431 (2.2)	467 (4.5)	757 (3.6)	870 (6.2)	802 (4.0)	863 (6.3)	496 (3.8)	524 (6.0)	159 (5.6)	149 (5.7)	17 (8.1)	10 (4.3)	< 0.001
Diabetes mellitus, *n* (%)	2058 (10.3)	1881 (18.0)	2581 (12.2)	2841 (20.2)	2374 (11.8)	2800 (20.5)	1594 (12.1)	1677 (19.3)	418 (14.8)	512 (19.7)	36 (17.1)	49 (20.9)	< 0.001
Dementia, *n* (%)	1358 (6.8)	615 (5.9)	3703 (17.5)	1449 (10.3)	5647 (28.1)	2380 (17.4)	5270 (40.0)	2372 (27.4)	1173 (41.4)	911 (35.1)	85 (40.5)	87 (37.0)	< 0.001
Hemiplegia, *n* (%)	259 (1.3)	175 (1.7)	566 (2.7)	422 (3.0)	367 (1.8)	395 (2.9)	213 (1.6)	222 (2.6)	64 (2.3)	73 (2.8)	3 (1.4)	6 (2.6)	< 0.001
Chronic kidney disease, *n* (%)	276 (1.4)	412 (3.9)	551 (2.6)	944 (6.7)	851 (4.2)	1171 (8.6)	707 (5.4)	882 (10.2)	286 (10.1)	356 (13.7)	35 (16.7)	30 (12.8)	< 0.001
COPD, *n* (%)	1960 (9.8)	1059 (10.1)	2475 (11.7)	1741 (12.4)	2227 (11.1)	1762 (12.9)	1401 (10.6)	1078 (12.4)	432 (15.3)	404 (15.6)	47 (22.4)	47 (20.0)	< 0.001
Connective tissue disease, *n* (%)	828 (4.1)	525 (5.0)	978 (4.6)	817 (5.8)	832 (4.1)	859 (6.3)	502 (3.8)	530 (6.1)	108 (3.8)	153 (5.9)	10 (4.8)	18 (7.7)	< 0.001
Peptic ulcer disease, *n* (%)	372 (1.9)	270 (2.6)	640 (3.0)	517 (3.7)	679 (3.4)	552 (4.0)	419 (3.2)	375 (4.3)	106 (3.7)	134 (5.2)	10 (4.8)	9 (3.8)	< 0.001
Liver disease, *n* (%)	261 (1.3)	163 (1.6)	256 (1.2)	223 (1.6)	149 (0.7)	114 (0.8)	59 (0.4)	51 (0.6)	19 (0.7)	13 (0.5)	1 (0.5)	2 (0.9)	< 0.001
Local cancer, *n* (%)	0 (0.0)	0 (0.0)	2205 (10.4)	1313 (9.3)	2479 (12.3)	1728 (12.6)	2097 (15.9)	1519 (17.5)	1189 (42.0)	886 (34.1)	181 (86.2)	208 (88.5)	< 0.001
Metastatic cancer, *n* (%)	0 (0.0)	0 (0.0)	631 (3.0)	242 (1.7)	705 (3.5)	299 (2.2)	398 (3.0)	209 (2.4)	147 (5.2)	114 (4.4)	29 (13.8)	27 (11.5)	< 0.001

*OFS* Orthopedic Hip Frailty Score, *BBt-* no beta-blocker therapy, *BBt+*  ongoing beta-blocker therapy, *ASA* American Society of Anesthesiologists, *OFS* Orthopedic Hip Frailty Score, *COPD* chronic obstructive pulmonary disease

The unadjusted 30-day postoperative mortality rate also increased as patients became more frail (BBt-, OFS 0 to 5: 1.8%, 6.2%, 12.2%, 18.4%, 30.8%, and 41.9%). Concurrently, despite generally being less fit for surgery as well as displaying a higher incidence of almost all comorbidities, BBt+ patients consistently exhibited a significantly lower postoperative mortality rate, compared to BBt- patients (BBt+ vs BBt-: OFS 0, 0.3% vs 1.8%; OFS 5, 17% vs 41.9%). This difference between the groups remained irrespective of the specific cause of mortality or stratum. Across all strata, the most common cause of mortality was cardiovascular events. This was also the mortality cause that exhibited the largest absolute reduction in all strata. However, all cause-specific mortality rates were lower in the BBt+ group compared to the BBt- group (Table 2).

**Table 2:** Crude outcomes in hip fracture patients

	OFS 0		OFS 1		OFS 2		OFS 3		OFS 4		OFS 5		
	BBt- (*N* = 20,003)	BBt+ (*N* = 10,460)	BBt- (*N* = 21,218)	BBt+ (*N* = 14,094)	BBt- (*N* = 20,113)	BBt+ (*N* = 13,685)	BBt- (*N* = 13,187)	BBt+ (*N* = 8,672)	BBt- (*N* = 2,831)	BBt+ (*N* = 2,597)	BBt- (*N* = 210)	BBt+ (*N* = 235)	*p* value
Length of stay, median [IQR]	7.0 [4.0–10]	8.0 [5.0–11]	9.0 [5.0–12]	9.0 [6.0–13]	8.0 [5.0–12]	9.0 [6.0–13]	7.0 [4.0–10]	8.0 [5.0–12]	6.0 [4.0–9.0]	7.0 [5.0–11]	6.0 [4.0–8.0]	6.0 [5.0–9.0]	< 0.001
Missing, *n* (%)	45 (0.2)	22 (0.2)	53 (0.2)	29 (0.2)	48 (0.2)	31 (0.2)	29 (0.2)	9 (0.1)	9 (0.3)	6 (0.2)	0 (0.0)	0 (0.0)	
30-day mortality, *n* (%)	351 (1.8)	29 (0.3)	1325 (6.2)	175 (1.2)	2451 (12.2)	448 (3.3)	2420 (18.4)	726 (8.4)	873 (30.8)	381 (14.7)	88 (41.9)	40 (17.0)	< 0.001
Cardiovascular event, *n* (%)	143 (0.7)	13 (0.1)	485 (2.3)	72 (0.5)	954 (4.7)	193 (1.4)	992 (7.5)	325 (3.7)	379 (13.4)	197 (7.6)	43 (20.5)	16 (6.8)	< 0.001
Respiratory failure, *n* (%)	84 (0.4)	3 (0.0)	276 (1.3)	28 (0.2)	425 (2.1)	69 (0.5)	372 (2.8)	103 (1.2)	133 (4.7)	43 (1.7)	12 (5.7)	6 (2.6)	< 0.001
Cerebrovascular event, *n* (%)	7 (0.0)	2 (0.0)	28 (0.1)	5 (0.0)	34 (0.2)	10 (0.1)	38 (0.3)	7 (0.1)	9 (0.3)	4 (0.2)	1 (0.5)	0 (0.0)	< 0.001
Sepsis, *n* (%)	7 (0.0)	1 (0.0)	28 (0.1)	5 (0.0)	43 (0.2)	6 (0.0)	42 (0.3)	16 (0.2)	22 (0.8)	5 (0.2)	2 (1.0)	1 (0.4)	< 0.001
Multiorgan failure, *n* (%)	100 (0.5)	9 (0.1)	460 (2.2)	62 (0.4)	909 (4.5)	148 (1.1)	863 (6.5)	246 (2.8)	311 (11.0)	113 (4.4)	30 (14.3)	16 (6.8)	< 0.001
Unknown, *n* (%)	10 (0.0)	1 (0.0)	48 (0.2)	3 (0.0)	86 (0.4)	22 (0.2)	113 (0.9)	29 (0.3)	19 (0.7)	19 (0.7)	0 (0.0)	1 (0.4)	< 0.001

Length of stay is measured in days

*OFS* Orthopedic Hip Frailty Score, *BBt-* no beta-blocker therapy, *BBt+*, ongoing beta-blocker therapy

After IPTW, all included potential confounders were balanced with an ASD < 0.1 (Supplemental Tables 1–6). BBt+ was associated with a reduced 30-day postoperative mortality rate, regardless of the degree of frailty. However, the adjusted ARR was larger in patients with higher frailty scores, increasing stepwise along with the OFS. Compared to BBt- patients, the mortality rate in BBt+ patients with OFS 0 was 2.2 percentage points lower [adjusted ARR (95% CI) 2.2 (2.0–2.4), *p* < 0.001], corresponding to a number needed to treat (NNT) of 45. Among BBt+ patients with OFS 5, the 30-day postoperative mortality rate was instead 24.1 percentage points lower [adjusted ARR (95% CI) 24.1 (18.3–30.0), *p* < 0.001] compared to BBt- patients, resulting in an NNT of 4. The sensitivity analysis, which also adjusted for the individual components of the OFS, resulted in virtually identical ARRs (Table 3).

**Table 3:** Absolute risk reduction in 30-day postoperative mortality rate in hip fracture patients, stratified by OFS

	Adjusted Absolute Risk Reduction (pp)	*p* value
OFS 0	2.2 (2.0–2.4)	< 0.001
OFS 1	6.0 (5.7–6.3)	< 0.001
OFS 2	10.1 (9.6–10.5)	< 0.001
OFS 3	10.7 (10.1–11.4)	< 0.001
OFS 4	15.6 (14.1–17.2)	< 0.001
OFS 5	24.1 (18.3–30.0)	< 0.001

The absolute risk reduction is defined as the absolute difference in mortality rate (%) between patients with and without beta-blocker therapy, after balancing the cohorts using inverse probability of treatment weighting. In each stratum cohorts were balanced based on age, sex, ASA classification, type of fracture, type of surgery, previous myocardial infarction, peripheral vascular disease, cerebrovascular disease, diabetes mellitus, dementia, chronic obstructive pulmonary disease, connective tissue disease, liver disease, and chronic kidney disease

*OFS* Orthopedic Hip Frailty Score, *ASA* American Society of Anesthesiologists

## Discussion

This Swedish nationwide cohort study, based on 127,305 cases, demonstrated an association between BBt and a reduction in postoperative mortality for all hip fracture patients. Using the OFS to stratify patients by the degree of frailty, a stepwise increase in the ARR associated with BBt was observed in conjunction with an increasing OFS. In effect, BBt was associated with a greater reduction in 30-day postoperative mortality rate the more frail a patient was, even after adjusting for differences in age, sex, fitness for surgery, fracture and surgery type, as well as comorbidity burden.

Hip fracture patients are one of the greatest challenges facing the orthopedic community and the postoperative mortality rates remain high despite efforts made during the past decade to reduce them [17, 53]. This has widely been attributed to old age, a significant comorbidity burden, and particularly the high degree of frailty present in this population [14–17]. However, in order to study frailty a clinically feasible tool that adequately measures frailty is required. The OFS is a parsimonious integral frailty model developed and validated for use in hip fracture patients [50, 54]. The inclusion of institutionalization and non-independent functional status allows for the consideration of the social, psychological, as well as cognitive aspects of frailty and are broadly accepted markers of frailty [55–57]. A history of malignancy and congestive heart failure on the other hand constitute comorbidities that reduce the patient’s physiological reserve and have therefore also been used as indicators of frailty [56–59]. Finally, while frailty and aging represent independent processes, the prevalence of frailty is recognized as increasing with age [35, 60, 61]. Moreover, an age ≥ 85 years on its own is insufficient for classifying a patient as frail with the OFS [50].

Frail patients, by definition, suffer from a disproportionately increased risk of morbidity and mortality due to external stressors [18–21]. Following hip fracture, the physiological stress response to this trauma, known as the hyperadrenergic state, is characterized by the activation of the sympathetic nervous system and mediated by a surge in catecholamines and cortisol. Despite being necessary for injury healing, this response can also have harmful side effects that can be disastrous in frail patients.These patients have a smaller margin for maintaining homeostasis as well as a reduced resistance to the catabolic state that is induced by the release of growth hormone, antidiuretic hormone, and glucagon [44, 62, 63].

It has been postulated that BBt could mitigate the hyperadrenergic state, through the binding of the active substance to adrenoreceptors, which inhibits the activation of adrenoreceptors by circulating catecholamines. Consequently, BBt is thought to reduce the negative effects caused by the physiological stress response [64]. Several studies have shown strong associations between BBt  and reduced mortality in a wide range of patient populations [38–42, 65–71]. A nationwide Swedish retrospective cohort study of patients undergoing elective colon cancer surgery, with an average patient age of 72, demonstrated an association between BBt and a 70% reduction in the 90-day postoperative mortality rate [adjusted Incident Rate Ratio (IRR) (95% CI) 0.29 (0.24–0.35), *p* < 0.001] [70]. Maghami et al. investigated this relationship in geriatric patients undergoing emergency laparotomies and found that patients with BBt exhibited a 35% reduction in their mortality rate [adjusted IRR (95% CI) 0.65 (0.44–0.98), *p* = 0.04] [71]. Furthermore, a randomized controlled trial found that postadmission administration of propranolol reduces in-hospital mortality and improves long-term functional outcomes in isolated severe traumatic brain injury [65]. All these studies claim the positive effects seen are due to downregulation of the toxic effects of the catecholamine surge.

The majority of the aforementioned studies include elderly and frail patients with multiple comorbidities, which mirrors the hip fracture population. Unsurprisingly, the same patterns have been seen in studies published during the last few years on hip fracture patients. A publication from 2021, demonstrated that BBt was associated with a 72% reduced mortality rate within 30 days after hip fracture surgery [adjusted IRR (95% CI) 0.28 (0.26–0.29), *p* < 0.001], where BBt+ was independently associated with a reduced risk of death due to cardiovascular, respiratory, and cerebrovascular events, as well as deaths due to sepsis or multiorgan failure [39]. These associations remained up to one year postoperatively [adjusted Hazard Ratio (95% CI) 0.58 (0.57–0.60), *p* < 0.001]. However, the largest effect appeared to be during the initial postoperative period, when the physiological stress response is most pronounced [40].

Considering that the chief causes of postoperative mortality after hip fracture surgery are of cardiovascular origin, in a previous study the authors investigated how the Revised Cardiac Risk Index (RCRI) affects the association between BBt and postoperative mortality. This study observed a stepwise reduction in mortality with a higher RCRI score for BBt+ patients, with the greatest reduction being observed in patients with the highest cardiac risk [41]. In a similar vein, the current results also found a stepwise relationship between BBt and frailty, where BBt was associated with a larger ARR in patients with a higher frailty score. Potentially because frail patients have a greater difficulty maintaining homeostasis when subjected to the posttraumatic hyperadrenergic state. Of note, while the prevalence of cardiovascular comorbidities does increase along with the OFS, Spearman’s rank correlation coefficient between the OFS and RCRI was < 0.3 in the current dataset. This negligible correlation indicates that the OFS correctly identifies frail cohorts that may derive an additional benefit of BBt rather than merely stratifying patients by cardiovascular risk [72].

Among these frail hip fracture patients, those with dementia can generally be considered to comprise a special subgroup [16, 73]. This disease can impede definitive diagnosis, hinder preoperative management and optimization, as well as reduce or eliminate compliance with postoperative treatment regiments. Approximately 20% of all hip fracture patients in Sweden have a diagnosis of dementia prior to suffering a hip fracture [16, 42]. An earlier study, based on a Swedish national sample population, observed that patients with dementia die from the same causes as other hip fracture patients, but to a much greater extent [16]. On the other hand, when investigating the association between BBt and mortality in patients with dementia undergoing hip fracture surgery, the 30-day postoperative mortality rate was reduced by 50% in those who had BBt at admission [adjusted IRR (95% CI) 0.50 (0.45–0.54), *p* < 0.001] [42]. Consequently, even this particularly vulnerable subgroup may benefit from BBt; however, a randomized controlled trial will be required to ascertain this definitively.

### Strengths and limitations

This analysis makes use of a large, national dataset consisting of over 127,000 consecutive hip fracture patients operated at a hospital in Sweden. The Rikshöft register this dataset is based on has a high case coverage, ranging between 80 and 90%, with 85% of hospitals that operate hip fractures contributing [74]. Accordingly, the register has received the highest certification level from the Swedish Association of Local Authorities and Regions [75, 76]. Loss to follow-up was also avoided through the use of the Swedish Cause of Death Register, which records all deaths within Sweden. Nevertheless, the usual caveats that limit the interpretability of all retrospective cohort studies, such as residual confounding and misclassification, must also be considered. It is also important to note that the BBt+ patients in the current study likely had a relatively well-adjusted BBt at admission. This does not automatically translate to a survival benefit when initiating BBt following admission due to hip fracture.

## Conclusion

Beta-blocker therapy is associated with a reduced 30-day postoperative mortality rate in frail hip fracture patients, with a greater effect being observed with higher Orthopedic Hip Frailty Scores.

## Supplementary Information

Below is the link to the electronic supplementary material.

Supplementary file1 (DOCX 46 KB)

Supplementary file2 (DOCX 33 KB)
