# Supplementary material for: A nationwide analysis on the interaction between frailty and beta-blocker therapy in hip fracture patients
Source: Eur J Trauma Emerg Surg. 2023 Jan 12;49(3):1485–97. doi: 10.1007/s00068-023-02219-7 (PMC10229461; doi:10.1007/s00068-023-02219-7)
Supplement: Supplementary file 1 — Supplementary file1 (DOCX 46 KB) [file 68_2023_2219_MOESM1_ESM.docx]

| **Supplemental Table 1.** Outcomes, demographics, and clinical characteristics of hip fracture patients with OFS 0, after inverse probability of treatment weighting | | | |
| --- | --- | --- | --- |
|  | **BBt- (N = 30,550)** | **BBt+ (N = 30,243)** | **ASD** |
| Length of stay, median [IQR] | 7 [4.0-11.0] | 7 [5.0-11.0] | 0.026 |
| 30-day mortality, n (%) | 735 (2.4) | 69 (0.2) | 0.192 |
| Age, median [IQR] | 75 [68.0-80.0] | 75 [68.0-80.0] | 0.026 |
| Sex, n (%) |  |  | 0.004 |
| Female | 19,913 (65.2) | 19,765 (65.4) |  |
| Male | 10,638 (34.8) | 10,478 (34.6) |  |
| ASA classification, n (%) |  |  | 0.018 |
| 1 | 4,282 (14.0) | 4,067 (13.4) |  |
| 2 | 16,073 (52.6) | 16,095 (53.2) |  |
| 3 | 9,476 (31.0) | 9,380 (31.0) |  |
| 4 | 708 (2.3) | 692 (2.3) |  |
| 5 | 11 (0.0) | 8 (0.0) |  |
| Type of fracture, n (%) |  |  | 0.009 |
| Non-displaced cervical (garden 1-2) | 4,914 (16.1) | 4,891 (16.2) |  |
| Displaced cervical (garden 3-4) | 12,103 (39.6) | 11,881 (39.3) |  |
| Basicervical | 947 (3.1) | 920 (3.0) |  |
| Peritrochanteric (two fragments) | 5,380 (17.6) | 5,335 (17.6) |  |
| Peritrochanteric (multiple fragments) | 4,557 (14.9) | 4,568 (15.1) |  |
| Subtrochanteric | 2,650 (8.7) | 2,648 (8.8) |  |
| Type of surgery, n (%) |  |  | 0.006 |
| Pins or screws | 6,795 (22.2) | 6,672 (22.1) |  |
| Screws or pins with sideplate | 7,090 (23.2) | 7,053 (23.3) |  |
| Intramedullary nail | 6,449 (21.1) | 6,397 (21.2) |  |
| Hemiarthroplasty | 4,760 (15.6) | 4,751 (15.7) |  |
| Total hip replacement | 5,457 (17.9) | 5,370 (17.8) |  |
| Previous myocardial infarction, n (%) | 851 (2.8) | 776 (2.6) | 0.013 |
| Peripheral vascular disease, n (%) | 938 (3.1) | 919 (3.0) | 0.002 |
| Cerebrovascular disease, n (%) | 3,048 (10.0) | 3,079 (10.2) | 0.007 |
| Dementia, n (%) | 1,972 (6.5) | 2,030 (6.7) | 0.010 |
| COPD, n (%) | 3,051 (10.0) | 3,101 (10.3) | 0.009 |
| Connective tissue disease, n (%) | 1,345 (4.4) | 1,353 (4.5) | 0.003 |
| Liver disease, n (%) | 437 (1.4) | 479 (1.6) | 0.013 |
| Diabetes mellitus, n (%) | 4,011 (13.1) | 4,031 (13.3) | 0.006 |
| Chronic kidney disease, n (%) | 731 (2.4) | 701 (2.3) | 0.005 |
| An ASD <0.1 is considered balanced.  OFS, Orthopedic Hip Frailty Score; BBt-, no beta-blocker therapy; BBt+, ongoing beta-blocker therapy; ASD, absolute standardized difference; ASA, American Society of Anesthesiologists; COPD, chronic obstructive pulmonary disease | | | |

| **Supplemental Table 2.** Outcomes, demographics, and clinical characteristics of hip fracture patients with OFS 1, after inverse probability of treatment weighting | | | |
| --- | --- | --- | --- |
|  | **BBt- (N = 35,441)** | **BBt+ (N = 35,187)** | **ASD** |
| Length of stay, median [IQR] | 9 [5.0-12.0] | 9 [6.0-13.0] | 0.058 |
| 30-day mortality, n (%) | 2,536 (7.2) | 416 (1.2) | 0.302 |
| Age, median [IQR] | 82 [76.0-87.0] | 82 [76.0-86.0] | 0.008 |
| Sex, n (%) |  |  | <0.001 |
| Female | 23,885 (67.4) | 23,722 (67.4) |  |
| Male | 11,555 (32.6) | 11,466 (32.6) |  |
| ASA classification, n (%) |  |  | 0.002 |
| 1 | 1,360 (3.8) | 1,358 (3.9) |  |
| 2 | 14,420 (40.7) | 14,322 (40.7) |  |
| 3 | 17,402 (49.1) | 17,284 (49.1) |  |
| 4 | 2,235 (6.3) | 2,202 (6.3) |  |
| 5 | 23 (0.1) | 22 (0.1) |  |
| Type of fracture, n (%) |  |  | 0.003 |
| Non-displaced cervical (garden 1-2) | 4,616 (13.0) | 4,591 (13.0) |  |
| Displaced cervical (garden 3-4) | 13,628 (38.5) | 13,527 (38.4) |  |
| Basicervical | 1,102 (3.1) | 1,092 (3.1) |  |
| Peritrochanteric (two fragments) | 7,077 (20.0) | 6,993 (19.9) |  |
| Peritrochanteric (multiple fragments) | 6,206 (17.5) | 6,192 (17.6) |  |
| Subtrochanteric | 2,812 (7.9) | 2,792 (7.9) |  |
| Type of surgery, n (%) |  |  | 0.007 |
| Pins or screws | 5,651 (15.9) | 5,646 (16.0) |  |
| Screws or pins with sideplate | 9,274 (26.2) | 9,135 (26.0) |  |
| Intramedullary nail | 7,924 (22.4) | 7,926 (22.5) |  |
| Hemiarthroplasty | 9,861 (27.8) | 9,795 (27.8) |  |
| Total hip replacement | 2,731 (7.7) | 2,685 (7.6) |  |
| Previous myocardial infarction, n (%) | 1,761 (5.0) | 1,662 (4.7) | 0.011 |
| Peripheral vascular disease, n (%) | 1,733 (4.9) | 1,685 (4.8) | 0.005 |
| Cerebrovascular disease, n (%) | 6,175 (17.4) | 6,164 (17.5) | 0.002 |
| Dementia, n (%) | 5,158 (14.6) | 5,087 (14.5) | 0.003 |
| COPD, n (%) | 4,268 (12.0) | 4,301 (12.2) | 0.006 |
| Connective tissue disease, n (%) | 1,809 (5.1) | 1,814 (5.2) | 0.002 |
| Liver disease, n (%) | 476 (1.3) | 493 (1.4) | 0.005 |
| Diabetes mellitus, n (%) | 5,527 (15.6) | 5,485 (15.6) | <0.001 |
| Chronic kidney disease, n (%) | 1,616 (4.6) | 1,517 (4.3) | 0.012 |
| An ASD <0.1 is considered balanced.  *OFS, Orthopedic Hip Frailty Score; BBt-, no beta-blocker therapy; BBt+, ongoing beta-blocker therapy; ASD, absolute standardized difference; ASA, American Society of Anesthesiologists; COPD, chronic obstructive pulmonary disease* | | | |

| **Supplemental Table 3.** Outcomes, demographics, and clinical characteristics of hip fracture patients with OFS 2, after inverse probability of treatment weighting | | | |
| --- | --- | --- | --- |
|  | **BBt- (N = 33,895)** | **BBt+ (N = 33,685)** | **ASD** |
| Length of stay, median [IQR] | 8 [5.0-12.0] | 9 [6.0-13.0] | 0.140 |
| 30-day mortality, n (%) | 4,559 (13.5) | 1,143 (3.4) | 0.368 |
| Age, median [IQR] | 87 [82.0-90.0] | 86 [82.0-90.0] | 0.003 |
| Sex, n (%) |  |  | 0.002 |
| Female | 23,586 (69.6) | 23,472 (69.7) |  |
| Male | 10,309 (30.4) | 10,213 (30.3) |  |
| ASA classification, n (%) |  |  | 0.006 |
| 1 | 670 (2.0) | 637 (1.9) |  |
| 2 | 10,636 (31.4) | 10,580 (31.4) |  |
| 3 | 19,266 (56.8) | 19,162 (56.9) |  |
| 4 | 3,286 (9.7) | 3,270 (9.7) |  |
| 5 | 36 (0.1) | 34 (0.1) |  |
| Type of fracture, n (%) |  |  | 0.003 |
| Non-displaced cervical (garden 1-2) | 4,218 (12.4) | 4,186 (12.4) |  |
| Displaced cervical (garden 3-4) | 12,303 (36.3) | 12,218 (36.3) |  |
| Basicervical | 1,182 (3.5) | 1,168 (3.5) |  |
| Peritrochanteric (two fragments) | 7,125 (21.0) | 7,083 (21.0) |  |
| Peritrochanteric (multiple fragments) | 6,394 (18.9) | 6,352 (18.9) |  |
| Subtrochanteric | 2,673 (7.9) | 2,678 (7.9) |  |
| Type of surgery, n (%) |  |  | 0.002 |
| Pins or screws | 5,257 (15.5) | 5,217 (15.5) |  |
| Screws or pins with sideplate | 9,138 (27.0) | 9,100 (27.0) |  |
| Intramedullary nail | 8,225 (24.3) | 8,174 (24.3) |  |
| Hemiarthroplasty | 10,195 (30.1) | 10,127 (30.1) |  |
| Total hip replacement | 1,080 (3.2) | 1,066 (3.2) |  |
| Previous myocardial infarction, n (%) | 2,475 (7.3) | 2,393 (7.1) | 0.008 |
| Peripheral vascular disease, n (%) | 1,778 (5.2) | 1,726 (5.1) | 0.005 |
| Cerebrovascular disease, n (%) | 6,756 (19.9) | 6,703 (19.9) | 0.001 |
| Dementia, n (%) | 8,007 (23.6) | 7,886 (23.4) | 0.005 |
| COPD, n (%) | 4,039 (11.9) | 4,039 (12.0) | 0.002 |
| Connective tissue disease, n (%) | 1,710 (5.0) | 1,697 (5.0) | <0.001 |
| Liver disease, n (%) | 281 (0.8) | 295 (0.9) | 0.005 |
| Diabetes mellitus, n (%) | 5,252 (15.5) | 5,216 (15.5) | <0.001 |
| Chronic kidney disease, n (%) | 2,130 (6.3) | 2,048 (6.1) | 0.008 |
| An ASD <0.1 is considered balanced.  *OFS, Orthopedic Hip Frailty Score; BBt-, no beta-blocker therapy; BBt+, ongoing beta-blocker therapy; ASD, absolute standardized difference; ASA, American Society of Anesthesiologists; COPD, chronic obstructive pulmonary disease* | | | |

| **Supplemental Table 4.** Outcomes, demographics, and clinical characteristics of hip fracture patients with OFS 3, after inverse probability of treatment weighting | | | |
| --- | --- | --- | --- |
|  | **BBt- (N = 21,938)** | **BBt+ (N = 21,772)** | **ASD** |
| Length of stay, median [IQR] | 7 [4.0-10.0] | 8 [5.0-12.0] | 0.186 |
| 30-day mortality, n (%) | 4,289 (19.5) | 1,920 (8.8) | 0.311 |
| Age, median [IQR] | 89 [86.0-92.0] | 89 [86.0-92.0] | 0.001 |
| Sex, n (%) |  |  | <0.001 |
| Female | 15,611 (71.2) | 15,495 (71.2) |  |
| Male | 6,327 (28.8) | 6,278 (28.8) |  |
| ASA classification, n (%) |  |  | 0.005 |
| 1 | 243 (1.1) | 235 (1.1) |  |
| 2 | 4,998 (22.8) | 4,948 (22.7) |  |
| 3 | 13,830 (63.0) | 13,765 (63.2) |  |
| 4 | 2,839 (12.9) | 2,798 (12.8) |  |
| 5 | 28 (0.1) | 27 (0.1) |  |
| Type of fracture, n (%) |  |  | 0.004 |
| Non-displaced cervical (garden 1-2) | 2,536 (11.6) | 2,515 (11.6) |  |
| Displaced cervical (garden 3-4) | 7,546 (34.4) | 7,462 (34.3) |  |
| Basicervical | 818 (3.7) | 821 (3.8) |  |
| Peritrochanteric (two fragments) | 4,947 (22.5) | 4,902 (22.5) |  |
| Peritrochanteric (multiple fragments) | 4,382 (20.0) | 4,370 (20.1) |  |
| Subtrochanteric | 1,710 (7.8) | 1,703 (7.8) |  |
| Type of surgery, n (%) |  |  | 0.002 |
| Pins or screws | 3,427 (15.6) | 3,390 (15.6) |  |
| Screws or pins with sideplate | 6,447 (29.4) | 6,404 (29.4) |  |
| Intramedullary nail | 5,487 (25.0) | 5,451 (25.0) |  |
| Hemiarthroplasty | 6,254 (28.5) | 6,204 (28.5) |  |
| Total hip replacement | 324 (1.5) | 323 (1.5) |  |
| Previous myocardial infarction, n (%) | 2,052 (9.4) | 1,990 (9.1) | 0.007 |
| Peripheral vascular disease, n (%) | 1,071 (4.9) | 1,040 (4.8) | 0.005 |
| Cerebrovascular disease, n (%) | 4,860 (22.2) | 4,840 (22.2) | 0.002 |
| Dementia, n (%) | 7,634 (34.8) | 7,583 (34.8) | 0.001 |
| COPD, n (%) | 2,532 (11.5) | 2,499 (11.5) | 0.002 |
| Connective tissue disease, n (%) | 1,036 (4.7) | 1,018 (4.7) | 0.002 |
| Liver disease, n (%) | 111 (0.5) | 112 (0.5) | 0.001 |
| Diabetes mellitus, n (%) | 3,339 (15.2) | 3,307 (15.2) | 0.001 |
| Chronic kidney disease, n (%) | 1,663 (7.6) | 1,612 (7.4) | 0.007 |
| An ASD <0.1 is considered balanced.  *OFS, Orthopedic Hip Frailty Score; BBt-, no beta-blocker therapy; BBt+, ongoing beta-blocker therapy; ASD, absolute standardized difference; ASA, American Society of Anesthesiologists; COPD, chronic obstructive pulmonary disease* | | | |

| **Supplemental Table 5.** Outcomes, demographics, and clinical characteristics of hip fracture patients with OFS 4, after inverse probability of treatment weighting | | | |
| --- | --- | --- | --- |
|  | **BBt- (N = 5,438)** | **BBt+ (N = 5,416)** | **ASD** |
| Length of stay, median [IQR] | 6 [4.0-9.0] | 7 [5.0-11.0] | 0.172 |
| 30-day mortality, n (%) | 1,684 (31.0) | 830 (15.3) | 0.378 |
| Age, median [IQR] | 90 [87.0-93.0] | 90 [87.0-93.0] | 0.003 |
| Sex, n (%) |  |  | 0.001 |
| Female | 3,478 (64.0) | 3,463 (63.9) |  |
| Male | 1,960 (36.0) | 1,953 (36.1) |  |
| ASA classification, n (%) |  |  | 0.004 |
| 1 | 22 (0.4) | 22 (0.4) |  |
| 2 | 647 (11.9) | 638 (11.8) |  |
| 3 | 3,673 (67.6) | 3,665 (67.7) |  |
| 4 | 1,075 (19.8) | 1,070 (19.8) |  |
| 5 | 20 (0.4) | 20 (0.4) |  |
| Type of fracture, n (%) |  |  | 0.004 |
| Non-displaced cervical (garden 1-2) | 639 (11.8) | 639 (11.8) |  |
| Displaced cervical (garden 3-4) | 1,829 (33.6) | 1,822 (33.6) |  |
| Basicervical | 201 (3.7) | 200 (3.7) |  |
| Peritrochanteric (two fragments) | 1,208 (22.2) | 1,202 (22.2) |  |
| Peritrochanteric (multiple fragments) | 1,108 (20.4) | 1,098 (20.3) |  |
| Subtrochanteric | 452 (8.3) | 455 (8.4) |  |
| Type of surgery, n (%) |  |  | 0.003 |
| Pins or screws | 952 (17.5) | 955 (17.6) |  |
| Screws or pins with sideplate | 1,499 (27.6) | 1,489 (27.5) |  |
| Intramedullary nail | 1,476 (27.1) | 1,471 (27.2) |  |
| Hemiarthroplasty | 1,457 (26.8) | 1,448 (26.7) |  |
| Total hip replacement | 54 (1.0) | 53 (1.0) |  |
| Previous myocardial infarction, n (%) | 752 (13.8) | 751 (13.9) | 0.001 |
| Peripheral vascular disease, n (%) | 317 (5.8) | 315 (5.8) | 0.001 |
| Cerebrovascular disease, n (%) | 1,451 (26.7) | 1,445 (26.7) | <0.001 |
| Dementia, n (%) | 2,087 (38.4) | 2,072 (38.3) | 0.003 |
| COPD, n (%) | 841 (15.5) | 847 (15.6) | 0.005 |
| Connective tissue disease, n (%) | 254 (4.7) | 257 (4.8) | 0.004 |
| Liver disease, n (%) | 32 (0.6) | 30 (0.6) | 0.003 |
| Diabetes mellitus, n (%) | 952 (17.5) | 942 (17.4) | 0.003 |
| Chronic kidney disease, n (%) | 662 (12.2) | 651 (12.0) | 0.005 |
| An ASD <0.1 is considered balanced.  *OFS, Orthopedic Hip Frailty Score; BBt-, no beta-blocker therapy; BBt+, ongoing beta-blocker therapy; ASD, absolute standardized difference; ASA, American Society of Anesthesiologists; COPD, chronic obstructive pulmonary disease* | | | |

| **Supplemental Table 6.** Outcomes, demographics, and clinical characteristics of hip fracture patients with OFS 5, after inverse probability of treatment weighting | | | |
| --- | --- | --- | --- |
|  | **BBt- (N = 443)** | **BBt+ (N = 446)** | **ASD** |
| Length of stay, median [IQR] | 6 [4.0-9.0] | 7 [5.0-9.0] | 0.050 |
| 30-day mortality, n (%) | 185 (41.9) | 79 (17.7) | 0.548 |
| Age, median [IQR] | 90 [87.0-93.0] | 90 [87.0-93.0] | 0.016 |
| Sex, n (%) |  |  | 0.021 |
| Female | 228 (51.4) | 225 (50.4) |  |
| Male | 215 (48.6) | 221 (49.6) |  |
| ASA classification, n (%) |  |  | 0.073 |
| 1 | 0 (0.0) | 1 (0.2) |  |
| 2 | 37 (8.3) | 35 (7.8) |  |
| 3 | 282 (63.8) | 288 (64.6) |  |
| 4 | 118 (26.5) | 116 (25.9) |  |
| 5 | 6 (1.4) | 6 (1.4) |  |
| Type of fracture, n (%) |  |  | 0.031 |
| Non-displaced cervical (garden 1-2) | 54 (12.2) | 57 (12.7) |  |
| Displaced cervical (garden 3-4) | 140 (31.7) | 139 (31.2) |  |
| Basicervical | 18 (4.1) | 20 (4.4) |  |
| Peritrochanteric (two fragments) | 101 (22.7) | 99 (22.1) |  |
| Peritrochanteric (multiple fragments) | 96 (21.7) | 96 (21.5) |  |
| Subtrochanteric | 34 (7.6) | 36 (8.0) |  |
| Type of surgery, n (%) |  |  | 0.028 |
| Pins or screws | 85 (19.2) | 84 (18.9) |  |
| Screws or pins with sideplate | 127 (28.7) | 127 (28.5) |  |
| Intramedullary nail | 121 (27.4) | 122 (27.4) |  |
| Hemiarthroplasty | 103 (23.3) | 107 (24.1) |  |
| Total hip replacement | 6 (1.4) | 5 (1.2) |  |
| Previous myocardial infarction, n (%) | 76 (17.1) | 75 (16.8) | 0.008 |
| Peripheral vascular disease, n (%) | 30 (6.7) | 30 (6.8) | 0.003 |
| Cerebrovascular disease, n (%) | 120 (27.1) | 121 (27.0) | 0.002 |
| Dementia, n (%) | 169 (38.2) | 171 (38.3) | 0.002 |
| COPD, n (%) | 98 (22.2) | 97 (21.7) | 0.012 |
| Connective tissue disease, n (%) | 25 (5.7) | 27 (6.0) | 0.014 |
| Liver disease, n (%) | 3 (0.8) | 3 (0.8) | <0.001 |
| Diabetes mellitus, n (%) | 86 (19.4) | 84 (18.9) | 0.013 |
| Chronic kidney disease, n (%) | 65 (14.6) | 67 (15.0) | 0.011 |
| An ASD <0.1 is considered balanced.  *OFS, Orthopedic Hip Frailty Score; BBt-, no beta-blocker therapy; BBt+, ongoing beta-blocker therapy; ASD, absolute standardized difference; ASA, American Society of Anesthesiologists; COPD, chronic obstructive pulmonary disease* | | | |
